# Whole shoot mineral partitioning and accumulation in pea (*Pisum sativum*)

**DOI:** 10.3389/fpls.2014.00149

**Published:** 2014-04-23

**Authors:** Renuka P. Sankaran, Michael A. Grusak

**Affiliations:** ^1^Department of Biological Sciences, Lehman College, City University of New YorkBronx, NY, USA; ^2^The Graduate School and University Center-City University of New YorkNew York, NY, USA; ^3^Department of Pediatrics, USDA/ARS Children's Nutrition Research Center, Baylor College of MedicineHouston, TX, USA

**Keywords:** PEA, mineral nutrition, remobilization, continuous uptake, seeds

## Abstract

Several grain legumes are staple food crops that are important sources of minerals for humans; unfortunately, our knowledge is incomplete with respect to the mechanisms of translocation of these minerals to the vegetative tissues and loading into seeds. Understanding the mechanism and partitioning of minerals in pea could help in developing cultivars with high mineral density. A mineral partitioning study was conducted in pea to assess whole-plant growth and mineral content and the potential source-sink remobilization of different minerals, especially during seed development. Shoot and root mineral content increased for all the minerals, although tissue-specific partitioning differed between the minerals. Net remobilization was observed for P, S, Cu, and Fe from both the vegetative tissues and pod wall, but the amounts remobilized were much below the total accumulation in the seeds. Within the mature pod, more minerals were partitioned to the seed fraction (>75%) at maturity than to the pod wall for all the minerals except Ca, where only 21% was partitioned to the seed fraction. Although there was evidence for net remobilization of some minerals from different tissues into seeds, continued uptake and translocation of minerals to source tissues during seed fill is as important, if not more important, than remobilization of previously stored minerals.

## Introduction

Plant foods are the principal source of dietary minerals for humans and animals. In particular, cereals such as rice, wheat, and maize, and grain legumes such as beans are the staple food in many populations. Moreover, seed quality is important because it may determine seedling vigor and help to increase crop yield (Pearson et al., 1995). The amount of minerals in the seeds depends on uptake from the soil into the roots, translocation into the shoots via the xylem, transfer into the leaves and other structures and translocation into the seeds via the phloem. Plant mineral concentrations also vary, depending on the species/cultivars and on the plant tissues, which emphasizes that genetic differences exist in the plants' ability to acquire and accumulate the minerals in different tissues. We have been interested in improving the mineral concentration of pea (*Pisum sativum*), an important grain legume that serves as an important source of nutrients for humans, especially in parts of the developing world (Cousin, 1997). Thus, we have conducted studies to develop a baseline understanding of whole-plant mineral content and dynamics in pea, as a first step toward subsequent efforts to improve seed mineral concentrations in this crop.

One of the main problems in trying to increase the nutrient content in plants is the lack of understanding of different pathways and gene products involved in transporting the minerals to the seeds. Several studies have identified genes/proteins involved in uptake of the minerals from the rhizosphere, some for translocation to vegetative tissues and ultimately accumulation in seeds (Grotz et al., 1998; Eren and Arguello, 2004; Green and Rogers, 2004; Hussain et al., 2004; Verret et al., 2004; Pittman, 2005; Andrés-Colás et al., 2006; Grotz and Guerinot, 2006; Colangelo and Guerinot, 2006; Durrett et al., 2007). Iron uptake has been shown to improve when ferric chelate reductase genes are overexpressed (Connolly et al., 2003; Vasconcelos et al., 2006). Pea (*brz* and *dgl*) and Arabidopsis (*frd3*) mutants with constitutive ferric reductase activity have been shown to accumulate various minerals such as Fe, Zn, Ca, Mg, Cu, and Mn in shoot tissues (Grusak, 2000; Rogers and Guerinot, 2002; Wang et al., 2003). Expression of the ferritin gene in rice has also resulted in an increased concentration of Fe in the seeds, but only to a small extent (10%) (Goto et al., 1999; Vasconcelos et al., 2003). Even though these studies have shown increased net mineral uptake in the plants, studies are still required to understand the spatial and temporal dynamics related to the movement of these minerals from vegetative tissues to the seeds, in order to achieve large increases in mineral concentrations in the seeds. There have been several studies addressing the partitioning of nitrogen in seeds and it has been established that remobilized N is a major source of seed protein components (Hortensteiner and Feller, 2002; Schiltz et al., 2005). There have also been some studies on partitioning and accumulation of other minerals in seeds which have shown that minerals may be remobilized from the vegetative tissues (Hocking and Pate, 1977; Himelblau and Amasino, 2001), but the data available are less abundant. Nonetheless, some studies have shown that continued mineral uptake and transport during seed fill is more important than remobilization, with respect to final mineral accumulation in seeds (Pate and Hocking, 1978; Waters and Grusak, 2008). Studies have also shown that nutrients can be translocated to the seeds via different tissues. However, information on these mineral dymanics is lacking, especially in important legume crops such as pea. In this paper, we describe the partitioning of nine different minerals (Ca, Mg, K, Cu, Fe, Mn, P, S, and Zn) prior to and during seed development in different tissues in pea (*Pisum sativum*), in order to assess the partitioning and the extent of remobilization of these minerals to the seeds.

## Materials and methods

Seeds of pea (*Pisum sativum* L., cv. Sparkle) were imbibed overnight in deionized water and grown hydroponically in aerated, buffered nutrient solution containing the following mineral nutrients: KNO_3_, 1.2 mM; Ca(NO_3_)_2_, 0.8 mM; NH_4_H_2_PO_4_, 0.3 mM; MgSO_4_, 0.2 mM; CaCl_2_, 25 μM; H_3_BO_3_, 25 μM; MnSO_4_, 2 μM; ZnSO_4_, 2 μM; CuSO_4_, 0.5 μM; H_2_MoO_4_, 0.5 μM; and NiSO_4_, 0.1 μM and were buffered with 1 mM MES [2-(N-morpholino) ethanesulfonic acid] buffer to maintain a pH between 5.5 and 6.0. Iron was added as Fe(III)-EDDHA (Grusak, 1994). Plants were grown in 3.5 L pots and the nutrient solutions were changed biweekly until reproductive maturity (10 weeks). Solution levels in the pots were checked daily and were adjusted to 3.5 L with nutrient solution as needed. All side shoots were excised at first appearance so as to yield a plant with only one main shoot. All plants were grown in a controlled environmental chamber with a 16 h, 20°C, and 8 h, 15°C day-night regime with a mixture of fluorescent and incandescent lights.

### Tissue analysis

Individual plants were harvested at weekly intervals, beginning at 2 weeks after planting, and were separated into seeds, pod walls, peduncles, flowers, and shoot remainder (main stem, stipules, and leaflets). Seeds from pods younger than 9 days after flowering were not separated from the pod walls. For flowers at a pre-anthesis state, or for post-anthesis flowers with non-emergent pod structures, the flower or flower bud was retained with the peduncle fraction. Note that because vegetative growth occurs throughout much of the plant's life cycle, and because flowers arise at sequential nodes up the stem, tissues of different developmental ages were combined at each harvest time point. Roots from weeks 4, 7, and 10 were included for mineral analysis. Roots from the other time points were not included for mineral analysis because they were used to measure the iron (III) reductase activity in a separate study (Grusak, 1995). Roots were rinsed in two changes of aerated deionized water, with each rinse lasting 2.5 min. They were then blotted dry and placed in paper bags for oven drying and subsequent dry weight determination. A total of 3–6 plants (replicates) were analyzed per time point.

All tissue samples were dried at 60°C to constant mass. Bulk tissues were homogenized with stainless steel grinders. For each sample, aliquots of 0.25 g of dried tissue sample were digested using 4 mL of concentrated nitric acid and 2 mL of perchloric acid at temperatures up to 200°C and then taken to dryness. Digests were resuspended in 1 mL 2 M HNO_3_ and, after 1 h, were brought to 10 mL with deionized water. The acids used were trace metal grade (Fisher Scientific, Pittsburgh, Pennsylvania, USA) and the water was deionized via a MilliQ system (Millipore, Billerica, Massachusetts, USA). Samples were analyzed for nine different minerals, Ca, Mg, K, Cu, Fe, Zn, Mn, S, and P using inductively coupled plasma-optical emission spectroscopy (CIROS ICP Model FCE12; Spectro, Kleve, Germany) to detect mineral concentrations, as previously described (Farnham et al., 2011).

Mineral content for each tissue was calculated for all nine minerals by multiplying each tissue's average mineral concentration by the average total tissue weight at a given time point. Net loss of minerals from the vegetative tissues during the reproductive phase was estimated by subtracting the final (week 10) mineral content in the non-seed tissues (leaves, stem, peduncles and pod wall) from the mineral content in the non-seed tissues during the beginning of the reproductive phase (week 5). To calculate the contribution of individual organs to seed mineral content (due to net remobilization), the mineral content of each tissue (at their peak level) was divided by the peak seed mineral content (week 10). For example, for Fe, the contribution of the vegetative tissues were based on the peak level (week 6) relative to peak seed mineral content at week 10.

### Statistics

Analyses of variance were performed using the JMP 10.0 program (SAS Institute, Cary, NC, USA). One Way ANOVA with Tukeys HSD was used to analyze the significance of differences in tissue mineral content across different time points and differences in mineral content between tissues. One Way ANOVA with Tukeys HSD was also used to calculate the difference between the different minerals in the percent of total minerals in seed fraction and the seed mineral content as percent of the total pod content (Figures 5, 6). Values after the “±” sign represent standard errors throughout the text.

## Results

**Figure 1:**
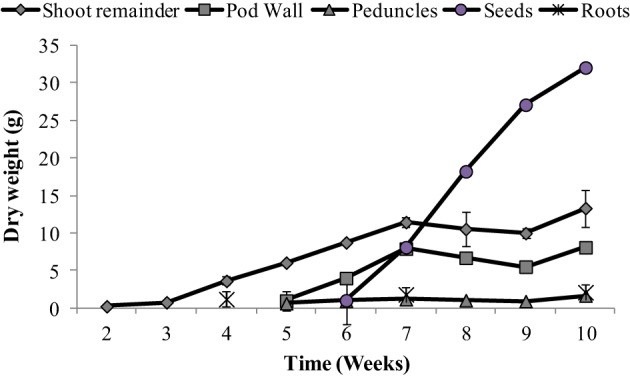
**Dry weight (g) of the different tissues (leaves + stem, pod, peduncle, and seeds) over time**. Values ± SE (*n* varies from 3 to 6). Some error bars do not extend beyond the symbols.

Organ-specific growth dynamics are presented in Figure 1. Plant DW was separated into the following components: seeds, pod walls, peduncles and flowers (referred to here as peduncles), and shoot remainder (main stem, stipules and leaflets). Seeds were the largest proportion of total shoot weight from weeks 8 through 10. Reproductive tissues (pod wall + seeds) represent 73% of the total shoot weight, while seeds represent 80% of the total reproductive tissue, at the final time point (Figure 1).

**Figure 2:**
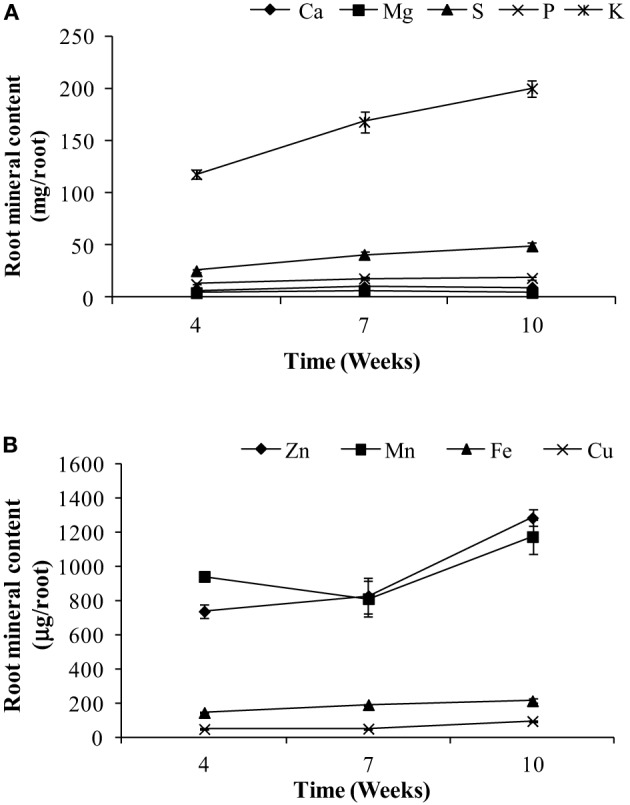
**Root content of (A) macronutrients and (B) micronutrients for weeks 4, 7, and 10 in pea**. Values ± SE (*n* varies from 3 to 6). Some error bars do not extend beyond the symbols.

**Figure 3:**
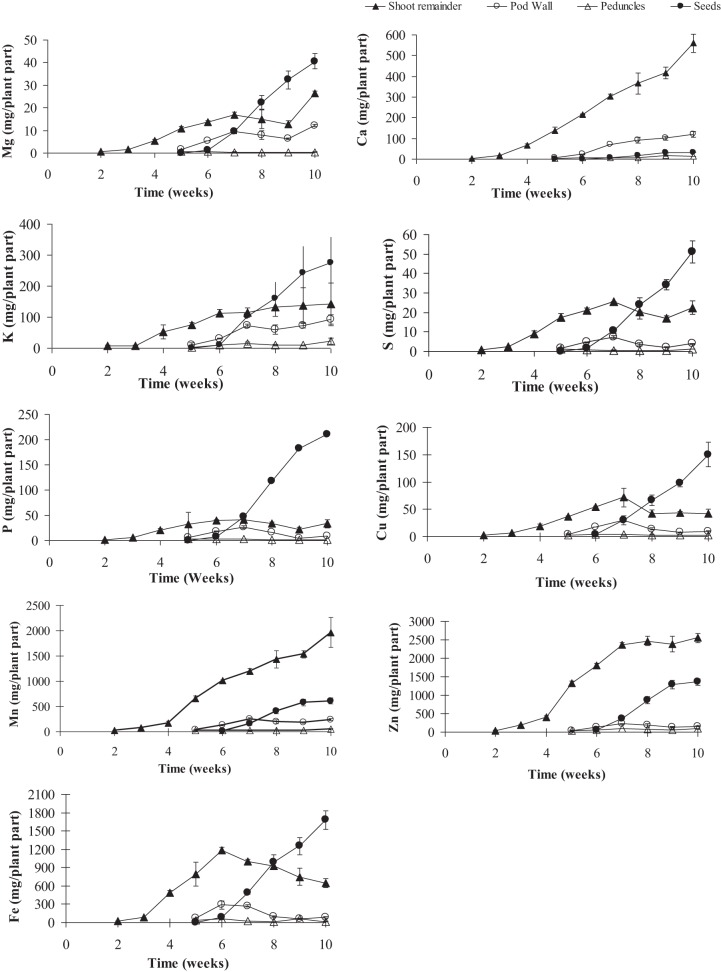
**Mineral contents of calcium (Ca), copper (Cu), iron (Fe), potassium (K), magnesium (Mg), manganese (Mn), phosphorus (P), sulfur (S), and zinc (Zn) in pea shoot tissues (leaves + stem, pod, peduncle, and seeds) over time**. Values ± SE (*n* varies from 3 to 6). Some error bars do not extend beyond the symbols (*p* ≤ 0.05).

**Figure 4:**
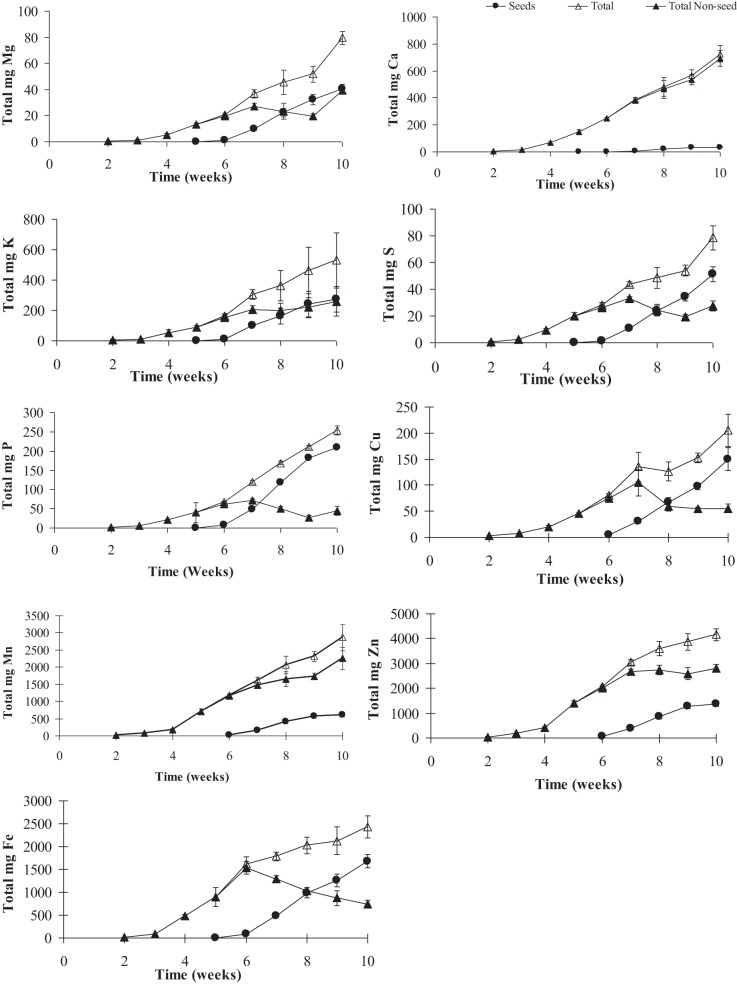
**Total mineral contents of calcium (Ca), copper (Cu), iron (Fe), potassium (K), magnesium (Mg), manganese (Mn), phosphorus (P), sulfur (S), and zinc (Zn) in pea shoots over time**. Values ± SE (*n* varies from 3 to 6). Some error bars do not extend beyond the symbols.

The main objective of this paper was to assess the whole-plant partitioning of different minerals to the seeds, especially the potential of the vegetative tissues to remobilize these minerals to the seeds. For this purpose, mineral content was calculated thoughout the plant's life cycle. Mineral content for different tissues are presented in Figures 2–4. For roots, we could only assess the mineral content at weeks 4, 7, and 10 due to the use of root tissues for a separate study, as mentioned above. Root weights are presented in Figure 1; average root weights increased from 1.19 ± 0.06 g DW (week 4), to 1.82 ± 0.09 g DW (week 7), to 2.12 ± 0.10 g DW (week 10). The root mineral content for most minerals increased or stayed constant over the time course (Figure 2), indicating that there was no net remobilization of minerals from the roots during the period of seed fill.

**Figure 5:**
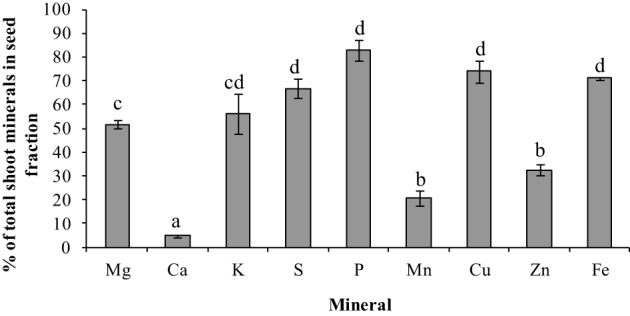
**Total mineral content in seed fraction as a percent of total shoot mineral content for the different minerals at seed maturity (week 10)**. Values ± SE (*n* varies from 3 to 6). Some error bars do not extend beyond the bars. Significant differences between the minerals is indicated by letters a–d (*p* ≤ 0.05).

To gauge the loss of minerals from the different tissues due to remobilization, we calculated the contribution of the different tissues based on their peak levels relative to the final seed mineral content at week 10. Overall, the total accumulation in the shoot tissue increased after the start of flowering (week 5). Net mineral content loss for some of the minerals and tissues was not evident and therefore could not be calculated. Mineral content of Ca, Mn, and Zn increased significantly in the vegetative tissues (leaves+stem) over the time course (Supplementary Table 1). For all other minerals, there was a significant decrease in mineral content in the vegetative tissues (between weeks 6 and 9) suggesting some amount of net remobilization from the tissues (*p* ≤ 0.05). There was also a significant decrease in the mineral content in the pod wall fraction over the time course for all the minerals except Ca, which suggests remobilization from the pod wall fraction (*p* ≤ 0.05) (Figures 3, 4). Total seed content of different minerals varied significantly between 4 and 82% of the total shoot mineral content at maturity (Figure 5). Among the macronutrients, only 4.6% of the shoot Ca was partitioned to the seeds which was significantly lower than all the other minerals, while 50% of the total shoot Mg and K was fractioned to the seeds and 65 and 82% of the total shoot S and P was fractioned to the seed tissues, respectively. Among the micronutrients, 21% of shoot Mn and 32% of shoot Zn was fractioned to the seeds, while significantly higher amounts of Fe and Cu (70–73%) were fractioned to the seeds (*p* ≤ 0.05) (Figure 5).

To assess the contribution of net remobilization from individual organs to seed mineral content, we have calculated the contribution of each of these individual tissues' peak levels relative to the peak seed mineral levels. For seed Ca, remobilization from the vegetative or pod tissue does not seem to be the source of seed Ca. For seed Mg, K, S, and Cu, remobilization from the vegetative tissues accounts for 50–60% of the total seed content while only 20% of the P from the vegetative tissues is fractioned into the seeds. For Fe, remobilization from the vegetative tissues is the highest among the minerals, accounting for 70% of the total seed Fe. There was no evidence of net remobilization from the vegetative tissues for Mn or Zn. Although starting with small pool sizes, all the minerals except Ca seem to remobilize from the pod wall and peduncles to the seeds. Remobilization from the pod wall fraction was the highest for Mn (40%) followed by K and Mg (30 and 34% respectively) while P, Cu, Fe, and Zn mobilized 20% or less of the total seed minerals. Remobilization from the pod wall fraction was the lowest for S at 9%. Remobilization from the peduncles accounted for less than 10% for all the minerals.

**Figure 6:**
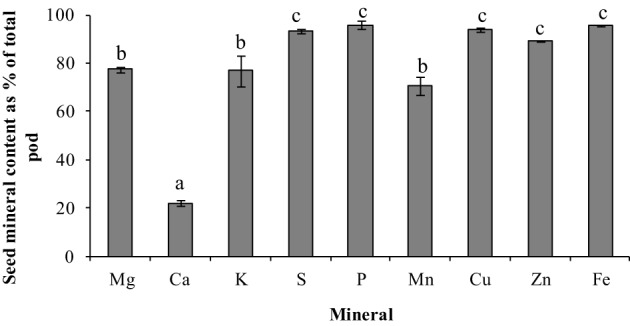
**Seed mineral content as percent of total pod mineral content for the different minerals in pea**. Values ± SE (*n* varies from 3 to 6). Some error bars do not extend beyond the bars. Significant differences between the minerals is indicated by letters a–c (*p* ≤ 0.05).

To understand the mineral distribution within the mature pod (pod wall + seed), the percentage of the total pod mineral content contained in seeds was calculated for each of the minerals (Figure 6). Except for Ca, the distribution pattern for all the minerals were similar, where more minerals were partitioned to the seed fraction than the pod wall (>75% of the total pod content). The seed mineral content for all the minerals was 75–95% of the total pod mineral content except for Ca, which was significantly lower where only 21% of the total fraction was partitioned to the seeds (*p* ≤ 0.05) (Figure 6). Also, the percent of minerals in the seeds that was taken up by the plant during the reproductive phase could be estimated only for Fe (90%). This might be because some of the incoming minerals will be partitioned to new leaves, stems, and roots.

## Discussion

Although there are a number of groups interested in increasing seed mineral concentrations in important plant foods such as rice, wheat, maize, and beans (Pfeiffer and McClafferty, 2007), our understanding of transport and remobilization of minerals to seeds is still incomplete. The goal of our study was to determine the main source of seed minerals in pea, and to analyze the potential of remobilization of previously stored minerals from various source tissues versus the continued uptake and translocation of minerals from the roots. Therefore, an increase in mineral content in one tissue can be either due to uptake and translocation from the soil into the plants, to the remobilization of minerals from one tissue to another, or both of these processes. In this study, we have measured changes in mineral content in different tissues in order to monitor the uptake, translocation and remobilization of the different minerals from one tissue to another, especially to seeds. We have also excised all side shoots to the point that we had only one main shoot.

Although remobilization from previously stored minerals in the vegetative tissues, such as leaves and roots has been studied previously (Hocking, 1994; Uauy et al., 2006), the contribution of remobilized minerals to seed mineral content is still unclear. The amounts of minerals that are being remobilized from the vegetative tissues will depend on the ability of the tissues to accumulate an excess of what is required locally in that tissue and also on the phloem mobility of each of the minerals. Remobilization from leaves has been observed for Cu, K, P, S, and Zn in two different studies in wheat (Hocking, 1994; Miller et al., 1994), while in another study there was very little remobilization of Zn from the vegetative tissues suggesting continuous uptake from the roots during seed fill (Garnett and Graham, 2005). In our study, among the macronutrients, mineral content of Mg, S, and P decreased in the vegetative tissues over time and could account for 20–65% of the total seed Mg, S, and P, suggesting remobilization from these tissues. Our study showed no net loss of Ca and K, (Figures 2, 3) consistent with studies in lupin, Arabidopsis and wheat (Hocking and Pate, 1977; Hocking, 1994; Himelblau and Amasino, 2001). Among the micronutrients, mineral content of Fe and Cu decreased in the vegetative tissues during the reproductive phase indicating remobilization of the minerals to the seeds. Remobilization of Fe and Cu from the pod wall fraction accounts for 48–70% of the total seed Fe and Cu. Previous studies have also shown remobilization of both Fe and Cu in wheat, beans, peas, and Arabidopsis (Grusak, 1995; Zhang et al., 1995; Himelblau and Amasino, 2001; Garnett and Graham, 2005; Waters and Grusak, 2008). In our study, there was no net remobilization observed from the vegetative tissues for Mn and Zn. Mn and Zn are known to have limited remobilization from the vegetative tissues. Consistent with our results, previous studies in Arabidopsis (Waters and Grusak, 2008), wheat (Pearson and Rengel, 1994) and clover (Nable and Loneragan, 1984; Himelblau and Amasino, 2001) for Mn and in wheat (Garnett and Graham, 2005) for Zn have also shown that they have limited mobility from the vegetative tissues, indicating continuous uptake from the root as the major source of these minerals in the seeds.

In addition to vegetative tissues, mineral loss from pod wall and peduncles could also account for the minerals being remobilized to the seeds. In a study with three legume species, Hocking and Pate (1977) showed that mobilization of minerals (N P, K, Ca, Mg, Fe, Cu, Mn, and Zn) from pods accounted for 5–39% of the total accumulation of specific seed minerals. In Arabidopsis, silique hulls have been shown to be a source of seed minerals (Waters and Grusak, 2008). In wheat, the Zn that was detected in different parts of the florets was detected in the grain (Pearson and Rengel, 1994). Although the pod wall pool is small to begin with, our results indicated that pod wall tissues are significant sources of seed minerals. Our results showed that 75–95% of the total pod (pod wall + seed) mineral content was partitioned to the seed tissue. The pod wall tissue contribution to the total seed mineral content varied between 9–40% for the different minerals, except for Ca where very little remobilization was observed from the pod wall tissues (Figure 6). Remobilization from the peduncles was also observed for all the minerals, although they contributed less than 10% of the total seed mineral content for most minerals. Although remobilization from the maternal fruit tissues is effective to increase the total seed mineral content, the plant would still require more total minerals coming in to the reproductive tissues to be partitioned to the seeds and increase the total seed mineral content.

In the present study, root mineral content was measured on weeks 4, 7, and 10. For all minerals, there was no evidence of a decline in mineral content during seed fill, demonstrating no net remobilization of any mineral to the seeds. Previous studies in wheat and Arabidopsis have shown that when minerals are readily available to the roots during the period of seed fill, the plants continue to take up those minerals and use them for seed mineral accumultion, suggesting that when sufficient nutrients are available in the soil, continuous uptake from the soil supersedes remobilization. However, in nutrient deficient conditions, minerals are remobilized from both root and shoot tissues (Peng and Li, 2005; Waters and Grusak, 2008; Waters et al., 2009).

In all of the studies mentioned above and in our present study, remobilization could not account for all of the seed minerals, indicating that plants continuously take up and translocate minerals to the seeds during the seed filling period. Although targeting enhanced remobilization of minerals from vegetative pools to seeds is a reasonable approach to increase seed mineral density, enhancing continuous uptake and translocation of minerals within the plant during seed fill is equally, if not more important, for increasing seed mineral density. Biofortification of pea will thus require a simultaneous enhancement of remobilization of minerals from different source tissues and more importantly will require targeting the processes involved in continuous uptake and translocation of minerals into seeds during seed development. Thus, understanding the genes responsible for both root uptake and for translocation may lead to strategies to produce biofortified seeds.

### Conflict of interest statement

The authors declare that the research was conducted in the absence of any commercial or financial relationships that could be construed as a potential conflict of interest.

## Supplementary material

Supplementary Table 1 **Mineral contents of pea tissues over time**. Means ± SE. Significant differences over time for each tissue is indicated by letters (a–f) (*P* ≤ 0.05).
